# Supplementary material for: A human cell-free translation screen identifies the NT-2 mycotoxin as a ribosomal inhibitor that binds the peptidyl transferase center
Source: Commun Biol. 2026 Sep 23;9:1237. doi: 10.1038/s42003-026-10743-6 (PMC13601500; doi:10.1038/s42003-026-10743-6)

Supplemental Information – Schwaller et al., 2026

A human cell-free translation screen identifies the NT-2 mycotoxin as a ribosomal inhibitor that binds the peptidyl transferase center

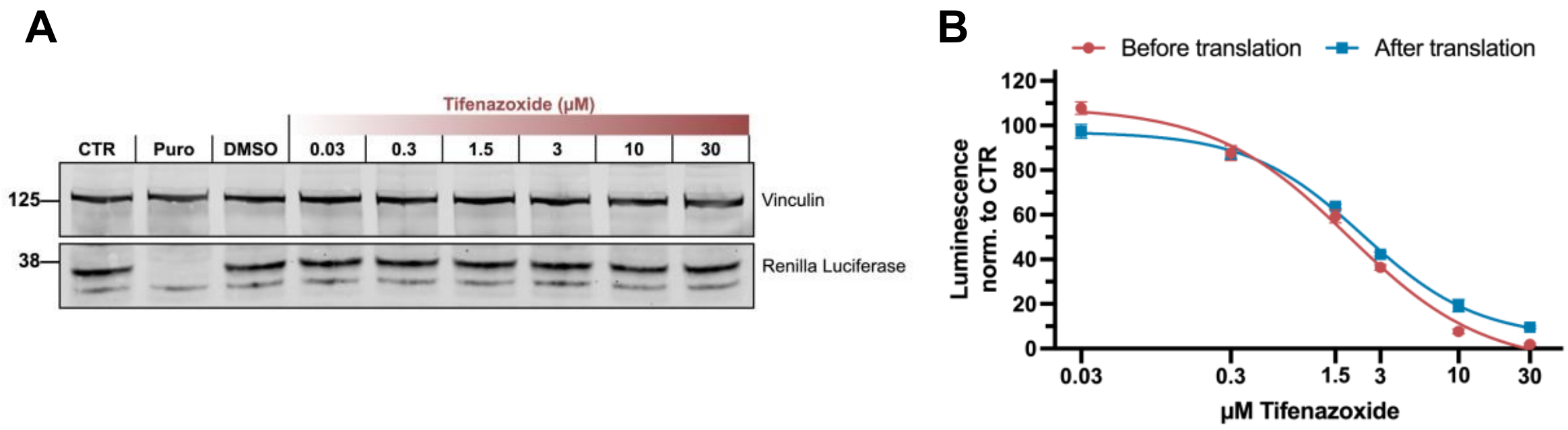

Supplementary Figure 1 | Characterization of Tifenazoxide as a *Renilla* luciferase inhibitor
(A) Western blot of an *in vitro* translation assay supplemented with tifenazoxide. Cell-free translation reactions were performed with *Renilla* luciferase mRNA in HeLa S3 lysate with increasing Tifenazoxide concentrations (30 nM - 30 μM) and were analyzed by SDS-PAGE and immunoblotting for *Renilla* luciferase (translation product) and Vinculin (loading control). **(B)** Cell-free translation reactions as described in (A) were performed with Tifenazoxide supplementation either before (red) or after (blue) translation. The measured luminescence was normalized to the negative control containing no supplement (CTR). Data are presented as mean values of three biological replicates (sets of translation reactions) averaged after three measurements ± SD.

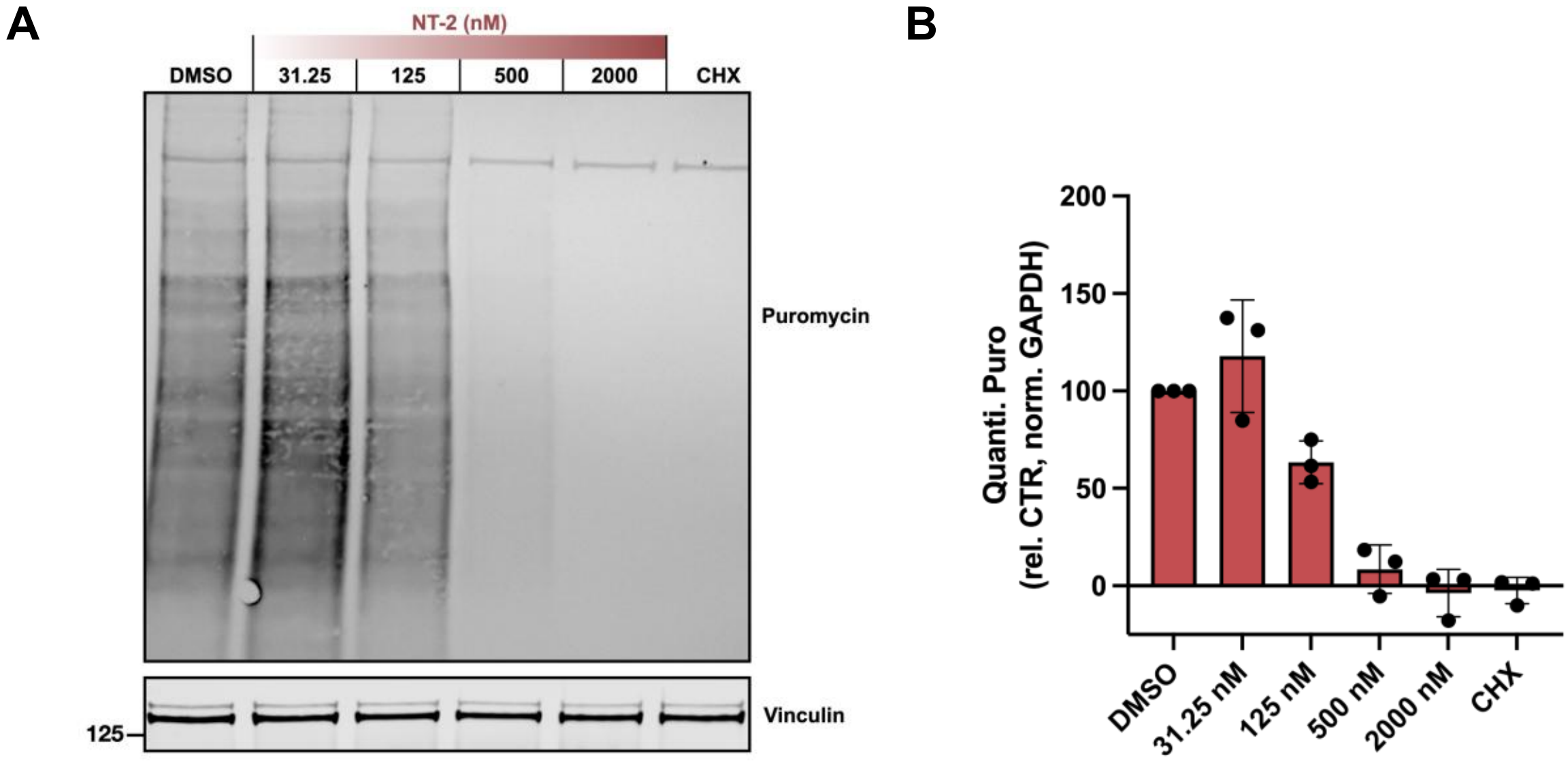

Supplementary Figure 2 | Effect of NT-2 on translation rates in *BHK-21 cells*

(A) Puromycin incorporation assay in BHK-21 cells treated with increasing NT-2 concentrations, DMSO, or CHX. Equal amounts of cell lysate were loaded into a 4-12% Bis-Tris gel and analysed by Western blot probing with anti-puromycin and anti-GAPDH antibodies. **(B)** Quantification of protein synthesis based on signal intensity in the anti-Puromycin-stained blot (Sup Fig 3A) normalized with GAPDH. Bars represent average of three biological replicates (dots) ± SD.

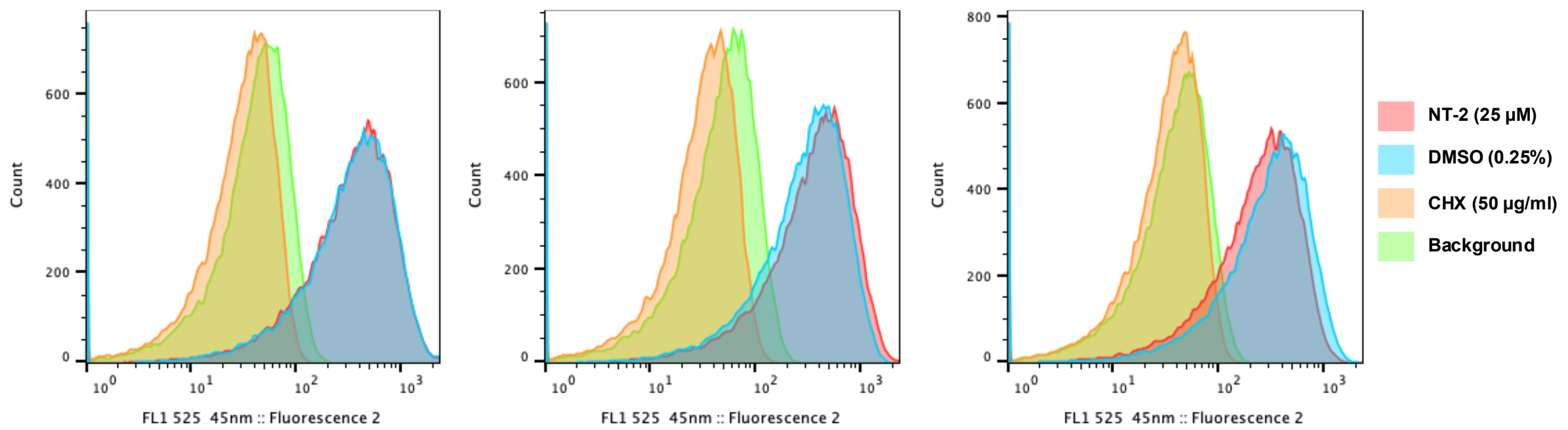

Supplementary Figure 3 | Effect of NT-2 on translation rates in *S. cerevisiae*
Fluorescence intensity of AHA-labeled yeast cells under different treatments. Cells were treated with NT-2 (25 µM), DMSO (0.25 %, control), CHX (50 µg/mL), or incubated in complete YPD medium (background control) and processed as described in Materials & Methods. Histograms show the distribution of single-cell fluorescence measured by flow cytometry of the three biological replicates.

NT-2 treated cells
44838 movies
drift correction
CTF fitting
denoising
↓
curation
42934 movies retained
↓
blob picker
3490267 particles
↓
extraction
960 px, binned to 96 px
2019542 particles
↓
2D classification
200 classes
1738165 particles retained
↓
ab initio model generation
heterogeneous refinement
3 classes
↓

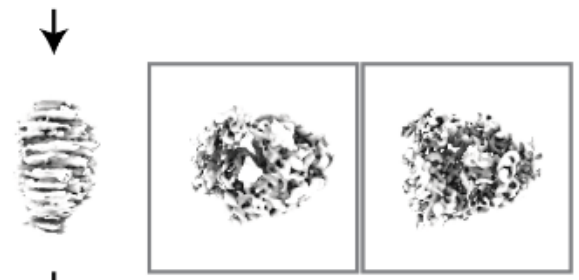

↓
homogeneous refinement
13.67 Å resolution
↓
extraction
960 px, binned to 720 px
1587552 particles
↓
homogeneous refinement
2.71 Å resolution
↓
global CTF refinement
↓
homogeneous refinement
2.06 Å resolution
↓
local CTF refinement
↓
homogeneous refinement
1.78 Å resolution
↓
reference motion correction
↓
homogeneous refinement
1.73 Å resolution
↓
global CTF refinement
↓
homogeneous refinement
1.72 Å resolution
→ downsampled to 640 px
↓
local refinement
1.77 Å resolution

DMSO control
7498 movies
drift correction, 4x bin
CTF fitting
denoising
↓
curation
7202 movies retained
↓
blob picker
372968 particles
↓
extraction
220 px, binned to 96 px
231399 particles
↓
2D classification
200 classes
186483 particles retained
↓
ab initio model generation
1 class
↓
extraction 256 px
↓
homogeneous refinement
5.29 Å resolution
↓
reference motion correction
↓
homogeneous refinement
3.25 Å resolution
↓
global CTF refinement
↓
homogeneous refinement
3.02 Å resolution
↓
downsampled to 640 px
↓
homogeneous refinement
3.0 Å resolution
↓
local CTF refinement
↓
homogeneous refinement
2.39 Å resolution
↓
local refinement
2.36 Å resolution

NT-2 control
26604 movies
drift correction, 4x bin
CTF fitting
denoising
↓
curation
23094 movies retained
↓
blob picker
1303310 particles
↓
extraction
220 px, binned to 96 px
809483 particles
↓
2D classification
200 classes
621588 particles retained
↓
ab initio model generation
1 class
↓
homogeneous refinement
12.53 Å resolution
↓
reference motion correction
↓
homogeneous refinement
3.11 Å resolution
↓
global CTF refinement
↓
homogeneous refinement
2.87 Å resolution
↓
downsampled to 640 px
↓
homogeneous refinement
2.80 Å resolution
↓
local CTF refinement
↓
homogeneous refinement
2.25 Å resolution
↓
local refinement
2.20 Å resolution

Supplementary Figure 4 | Cryo-EM data processing flowcharts.
Image processing workflows for the three datasets: 80S ribosomes isolated from NT-2–treated cells, 80S isolated from DMSO-treated (CTR) cells and NT-2–incubated 80S ribosomes. Flowcharts summarize particle selection, classification, and refinement steps leading to the final reconstructions.

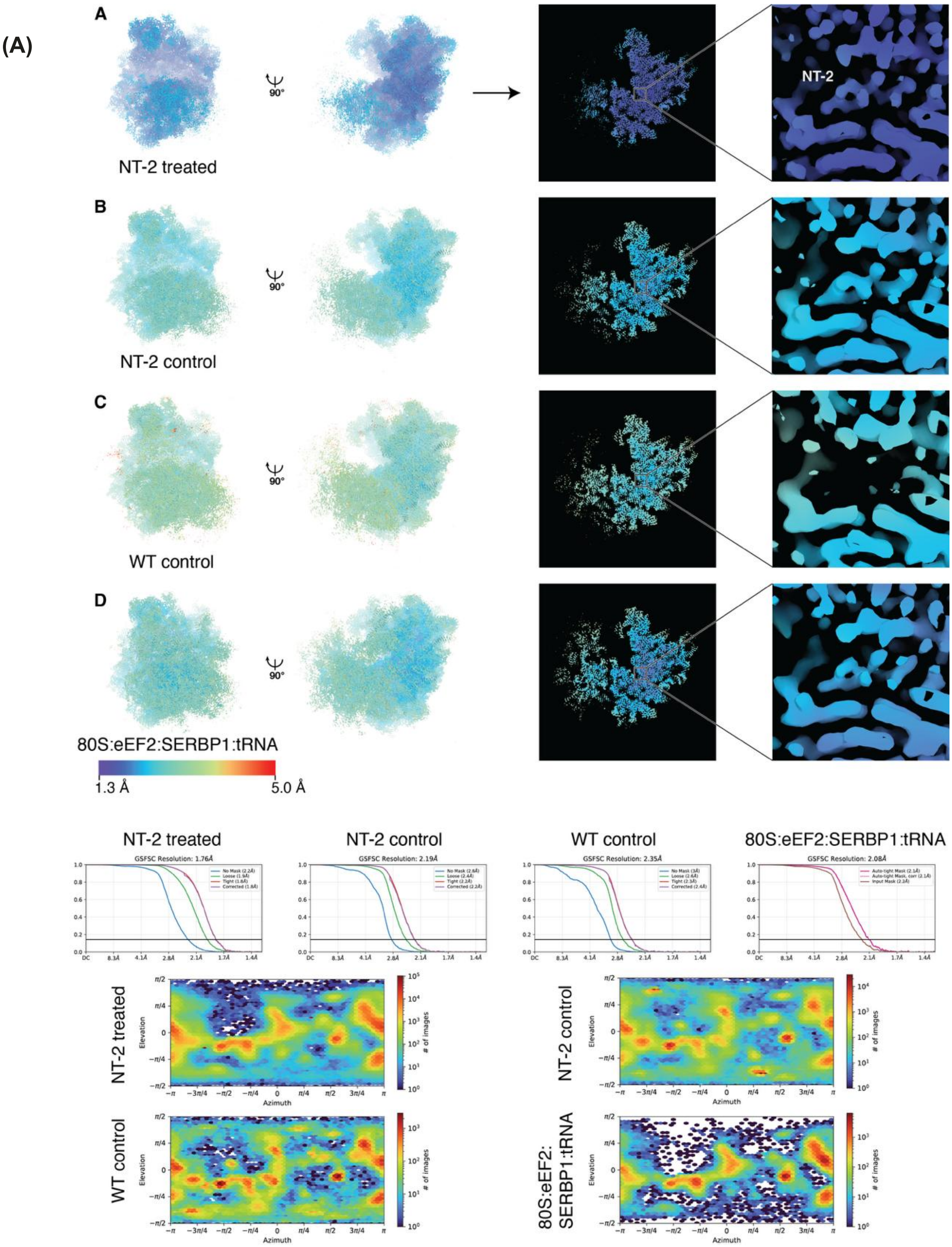

Supplementary Figure 5 | Resolution maps, FSC curves and particle orientation distribution plots. Resolution map of monosomes from NT-2 treated cells, focused on the 60S subunit. A resolution map of the surface of the molecule is shown on the left two panels. The center right panel shows a section through the map around the binding site of NT-2. On the right panel, NT-2 and the region directly adjacent are shown. All resolution maps are shown on the same scale (see inset below panel D). **(B)** Resolution map of purified monosomes incubated with NT-2 before vitrification, focused on the 60S subunit. **(C)** Resolution map of purified monosomes, focused on the 60S subunit. NT-2 is absent from the binding site. **(D)** Resolution map of dormant 80S:eEF2:SERBP1:tRNA complex purified from NT-2-treated cells. **(E)** Fourier shell correlation curves. F) Particle distribution plots.

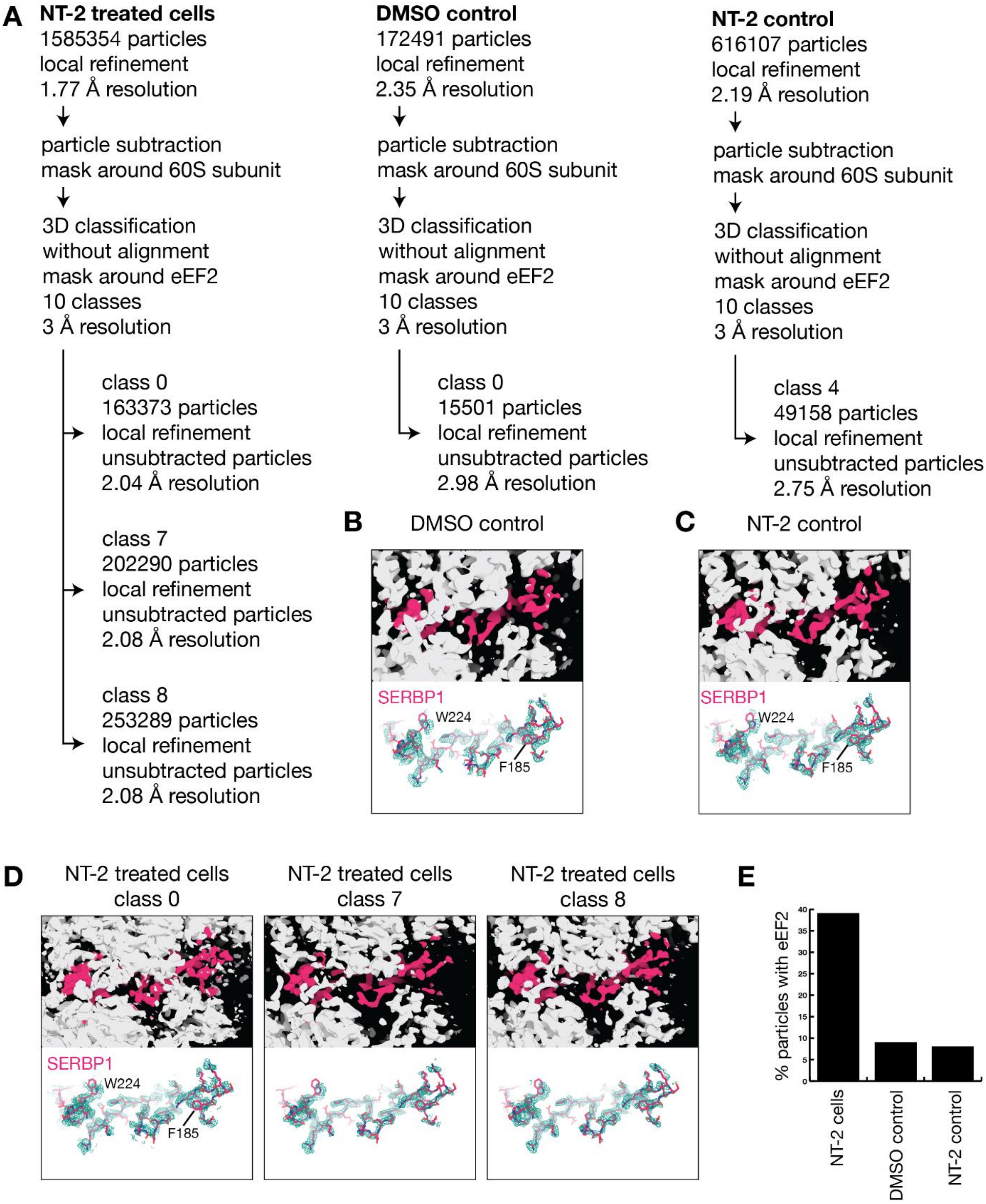

Supplementary Figure 6| Investigation of eEF2-bound 80S complexes by focused classification. **(A)** Schematic representation of the processing workflow. **(B)** Observed Cryo-EM density for the DMSO control. Ribosome density is shown as white surface and density corresponding to SERBP1 is shown in magenta (top). A rigid-body fitted atomic model of SERBP1 is overlayed with the experimental density of SERBP1 in the DMSO control (bottom). **(C)** Observed Cryo-EM density for the NT-2 control **(D)** Observed Cryo-EM density for the NT-2 treated cells. Density from three classes is shown; all classes contain bound SERBP1. **(E)** Comparison of class sizes after focused classification. While 39% of ribosomes from NT-2 treated cells are found in the dormant SERBP1 and eEF2-bound 80S state, only 9% and 8%, respectively of control ribosomes adopt this state.

Sup. Table 1 | Primary screening reproducibility:

Standard deviation of replicates per compound across plates:

Parameter	Prestwick	Repurposing	PPI	NP inspired	NP	CDC
Mean SD	0.066	0.041	0.053	0.051	0.045	0.049
SD of SD	0.053	0.032	0.056	0.040	0.038	0.050

Coefficient of variation of primary hits:

Parameter	Value (%)
Mean CV	4.588
SD CV	4.896

Sup. Table 2 | DRC's secondary hits:

Compound Name	CAS	Library	*In vitro*: inhibits…		*In vivo* active?
			Renilla luciferase	Translation	
NT 2 Toxin	76348-84-0	Natural Product		X	*Yes*
F3292-0451	306958-80-5	NP-inspired		X	*No*
Mitoxantrone dihydrochloride	70476-82-3	Prestwick		X	*No*
RA-XII	143343-98-0	Natural Product		X	*No*
UNC0224	1197196-48-7	Repurposing		X	*No*
Alizapride HCl	59338-87-3	Prestwick	X		
BIX-01294	1392399-03-9	Repurposing	X		
GR46611	185259-85-2	Repurposing	X		
Khasianine	32449-98-2	Natural Product	X		
Mdivi-1	338967-87-6	Repurposing	X		
PI4KIIIbeta-IN-10	1881233-39-1	Repurposing	X		
Prenylamine lactate	69-43-2	Prestwick	X		
Solamargine	20311-51-7	Repurposing	X		
Sulfaquinoxaline sodium salt	967-80-6	Prestwick	X		
tifenazoxide	279215-43-9	Repurposing	X		
Walrycin B	878419-78-4	Repurposing	X		
ZCL278	587841-73-4	Repurposing	X		

Sup. Table 3 | Known translation inhibitors in DRC's secondary hits:

Compound Name	CAS	Library
15-Monoacetoxyscirpenol	2623-22-5	Natural Product
Anguidine	2270-40-8	Natural Product
Anisomycin	22862-76-6	Repurposing
Cycloheximide	66-81-9	Prestwick
Emetine (dihydrochloride hydrate)	316-42-7	Repurposing
Harringtonine	26833-85-2	Repurposing
Homoharringtonine	26833-87-4	Repurposing
Hygromycin B	31282-04-9	Repurposing
Puromycin (dihydrochloride)	58-58-2	Repurposing
T2 toxin	21259-20-1	Natural Product

Sup. Table 4 | DRC's secondary hits without further characterization:

Compound Name	CAS	Library
2,4,7,9-tetramethylbenzo[b][1,8]naphthyridin-5-amine	309726-06-5	CDC
3-(2-chlorophenyl)-1-ethyl-6-methylpyrimido[5,4-e][1,2,4]triazine-5,7(1H,6H)-dione	891030-35-6	CDC
3-(phenylsulfonyl)-2-piperazino-6,7,8,9-tetrahydropyrazolo[1,5-a]pyrido[3,4-e]pyrimidine	1194864-48-6	CDC
3,7-dimethyl-2,6-acridinediamine	931328-91-5	CDC
4-{[3-(diethylamino)propyl]amino}benzo[g]quinolinium-1-olate	n.a	CDC
5-{[3-(dimethylamino)propyl]amino}-9,10-dimethyl-8H-thieno[2',3':4,5]pyrimido[2,1-a]phthalazin-8-one	380455-04-9	CDC
Bruceantin	41451-75-6	Repurposing
Ethyl 3-[[[(4-fluorophenyl)methyl]amino]sulfonyl]benzo[b]thiophene-2-carboxylate	941962-39-6	PPI
Halofuginone	55837-20-2	Repurposing
Hemin	16009-13-5	Repurposing
4-(2-(3,5-dimethyl-2-oxocyclohexyl)-2-hydroxyethyl)piperidine-2,6-dione	17280-60-3	Natural Product
Rubiadin-primveroside	26388-47-6	Natural Product
Solamargine derivative (catalogID NP-014212)	n.a	Natural Product
(2R,2′S,3R,5R,5aR,7S,9aR)-5a-[(Acetyloxy)methyl]-2,3,4,5,5a,6,7,9a-octahydro-3-hydroxy-5,8-dimethylspiro[2,5-methano-1-benzoxepin-10,2′-oxiran]-7-yl 3-methylbutanoate	1471987-91-3	Natural Product
Propidium iodide	25535-16-4	Prestwick
RA-XIII	143277-28-5	Natural Product
(-)-Emetine	483-18-1	Natural Product
T 2 Toxin derivative (catalogID STOCK1N-56995)	n.a	Natural Product
Lycorine	476-28-8	Natural Product

Sup. Table 5 | Structural comparison of trichothecenes in screen:

CatalogID	Name	Class	R1	R2	R3	R4	R5	SCORE
NP-004914	8-Hydroxyisotrichodermin	A	OAc	H	H	H	OH	0.479
NP-002481	Anguidine	A	OH	OAc	OAc	H	H	0.843
NP-015158	NT2 Toxin	A	OH	OAc	OH	H	OH	0.735
NP-002478	Neosolaniol	A	OH	OAc	OAc	H	OH	0.584
NP-019349	Toxin T2 tetrol	A	OH	OH	OH	H	OH	0.594
NP-002684	15-Monoacetoxy scirpenol	A	OH	OH	OAc	H	H	0.865
STOCK1N-56995	T2 -Toxin	A	OH	OAc	OAc	H	$OCOCH_2CH(CH_3)_2$	1.001
NP-019199	-	A	OH	H	OAc	H	$OCOCH_2CH(CH_3)_2$	0.682
NP-019350	4-Deacetylneosolaniol	A	OH	OAc	OAc	H	OH	0.393
NP-004848	Acetyldeoxynivalenol	B	OAc	H	OH	OH	O =	0.371
NP-004844	Deoxynivalenol	B	OH	H	OH	OH	O =	0.276

Uncropped Western Blot Raw Images

Fig. 3D FLAG Antibody

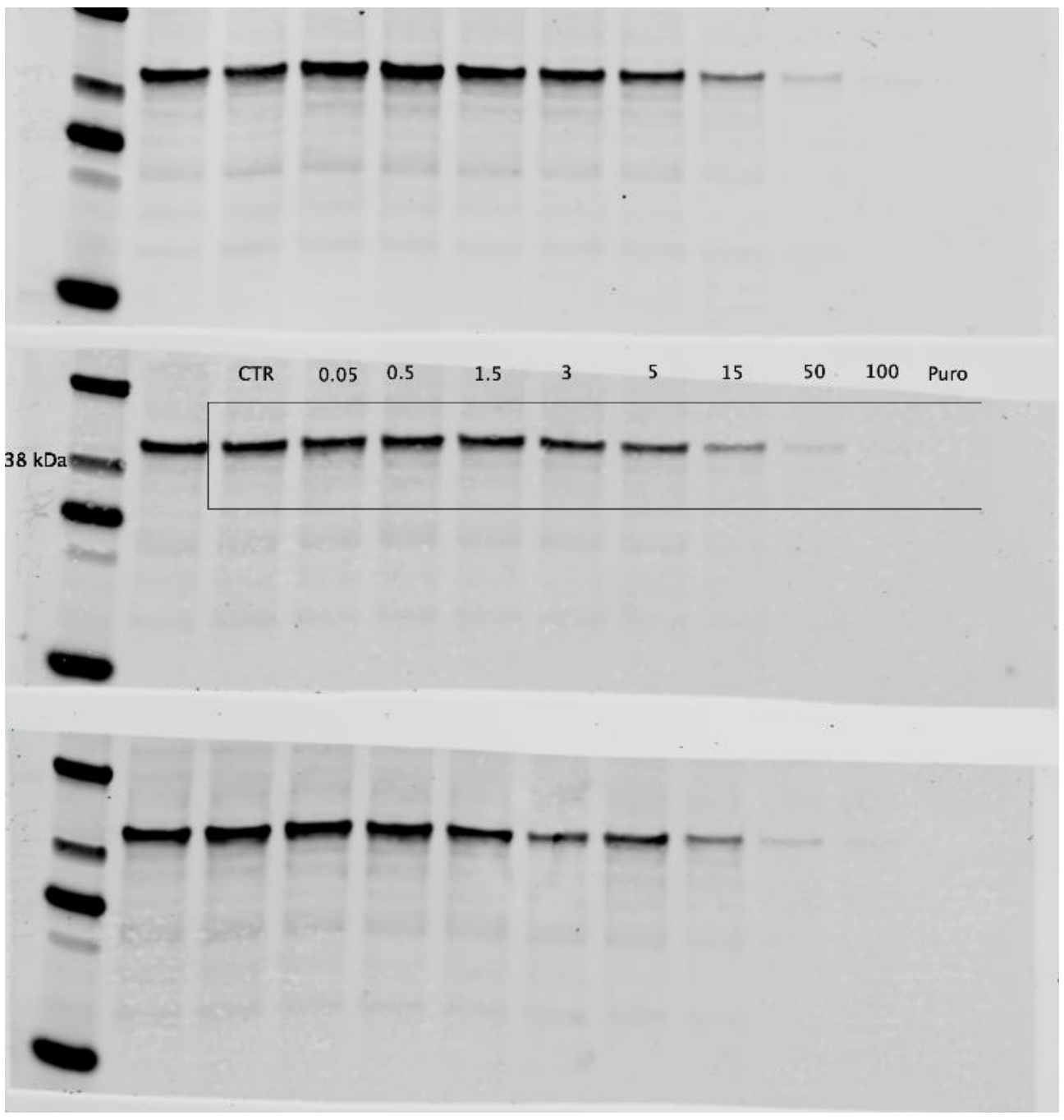

Figure 3D Vinculin Antibody

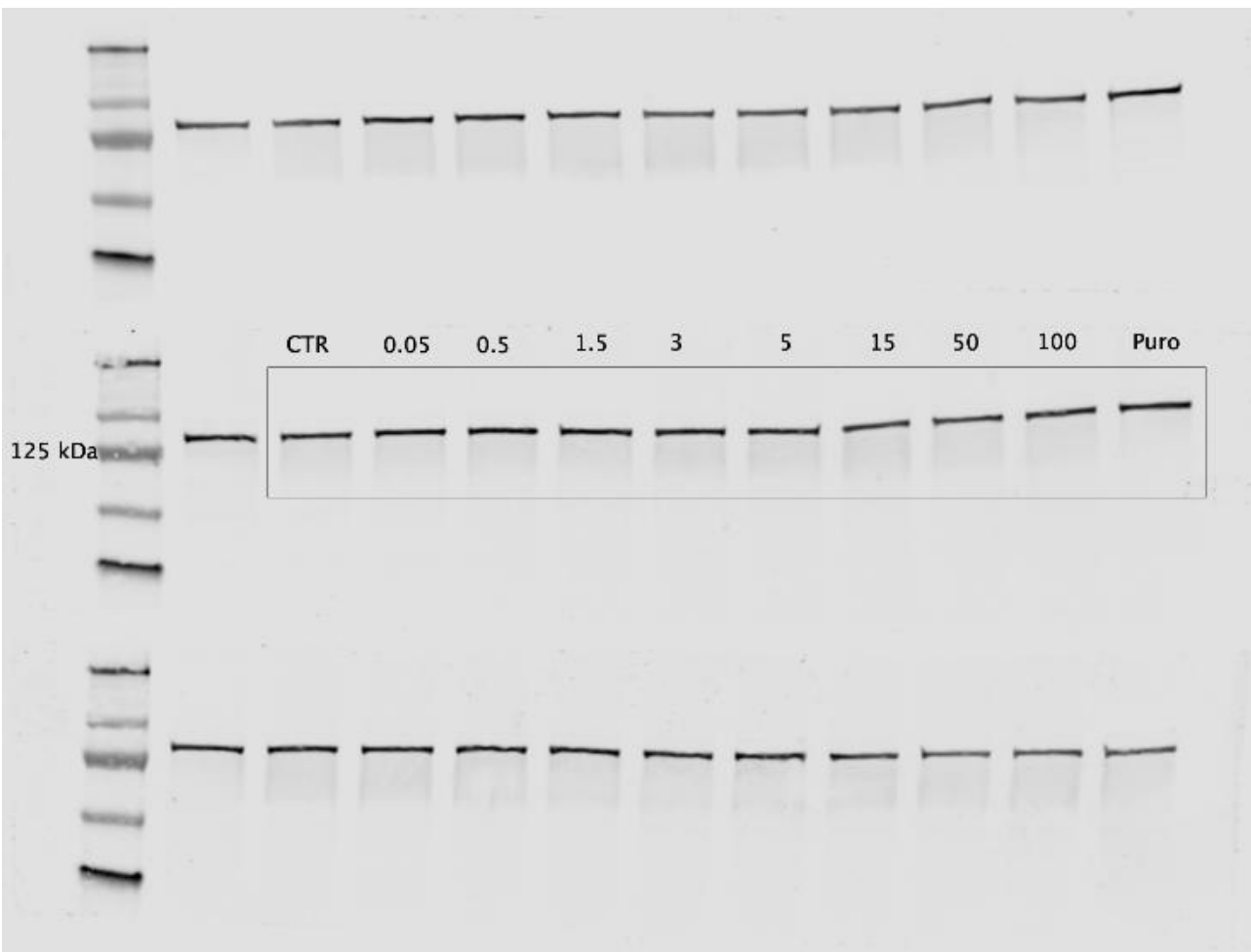

Figure 4A GAPDH Antibody

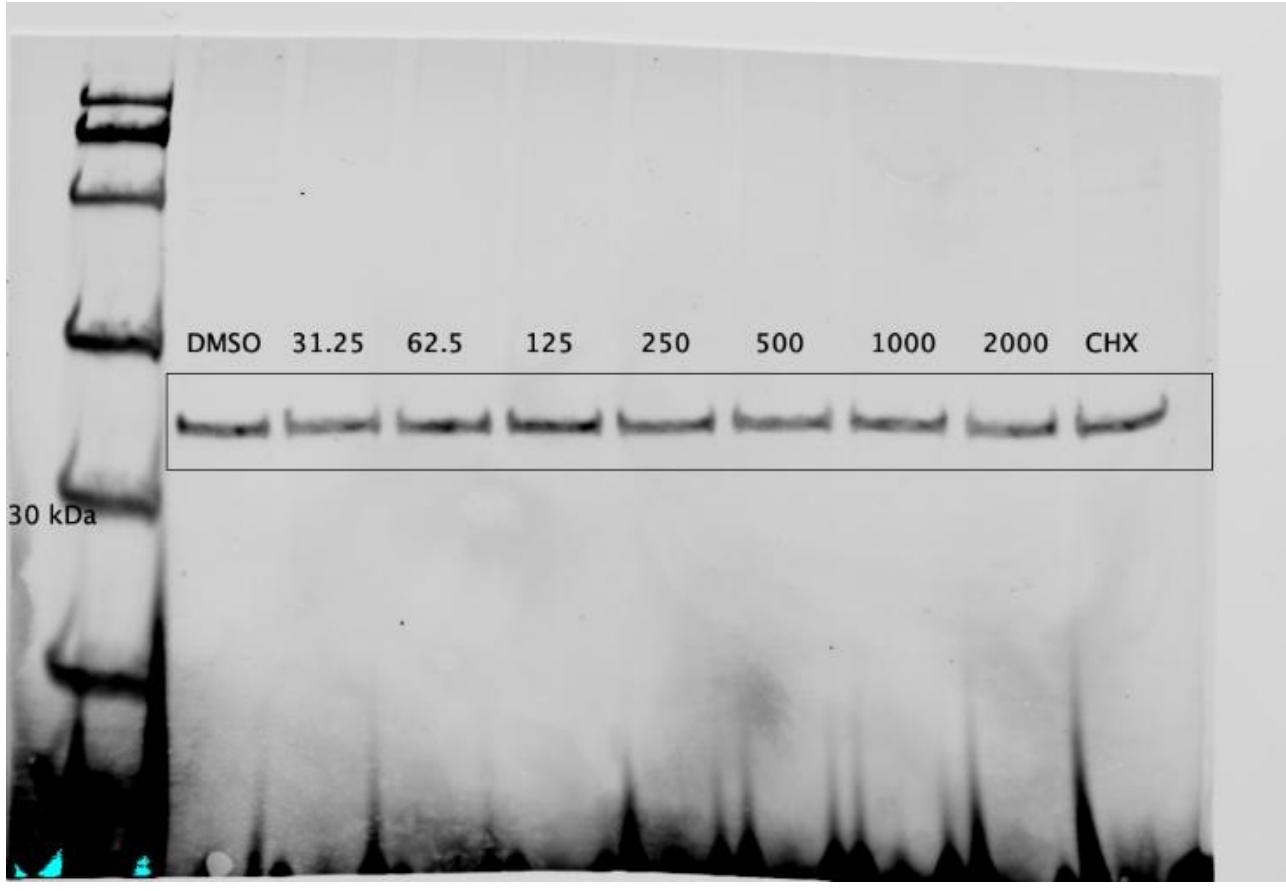

Figure 4A Puromycin Antibody

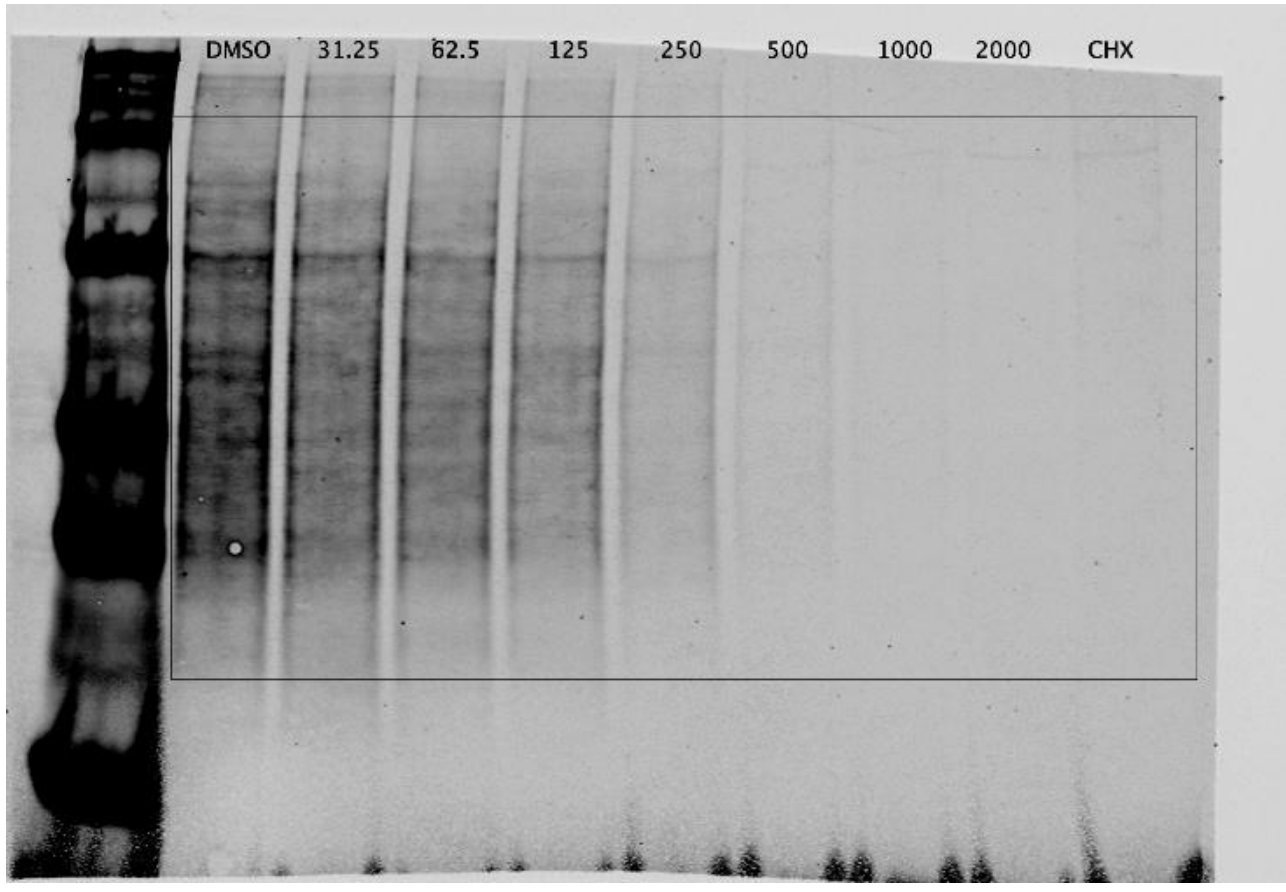

Figure 4D phospho-eiF2alpha Antibody

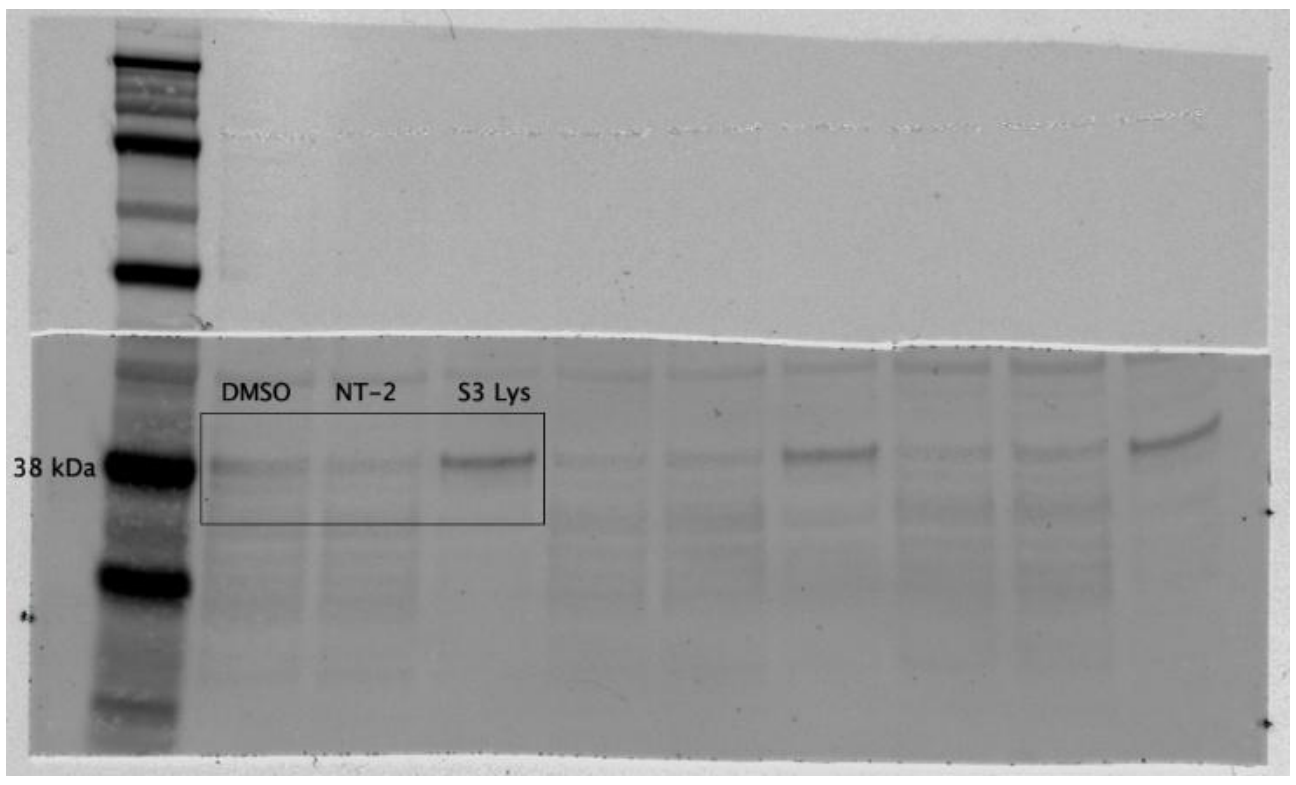

Figure 4D Vinculin & eIF2alpha Antibodies

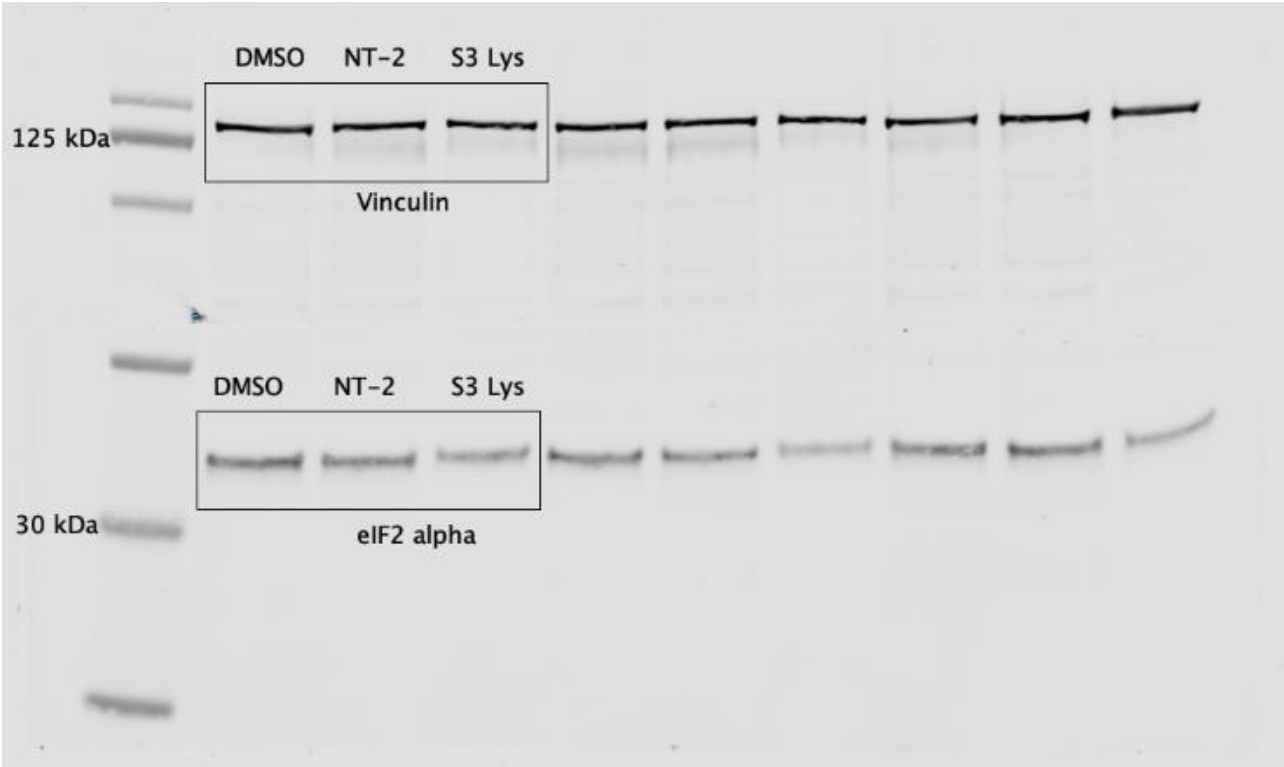

Sup. Figure 1A Luciferase

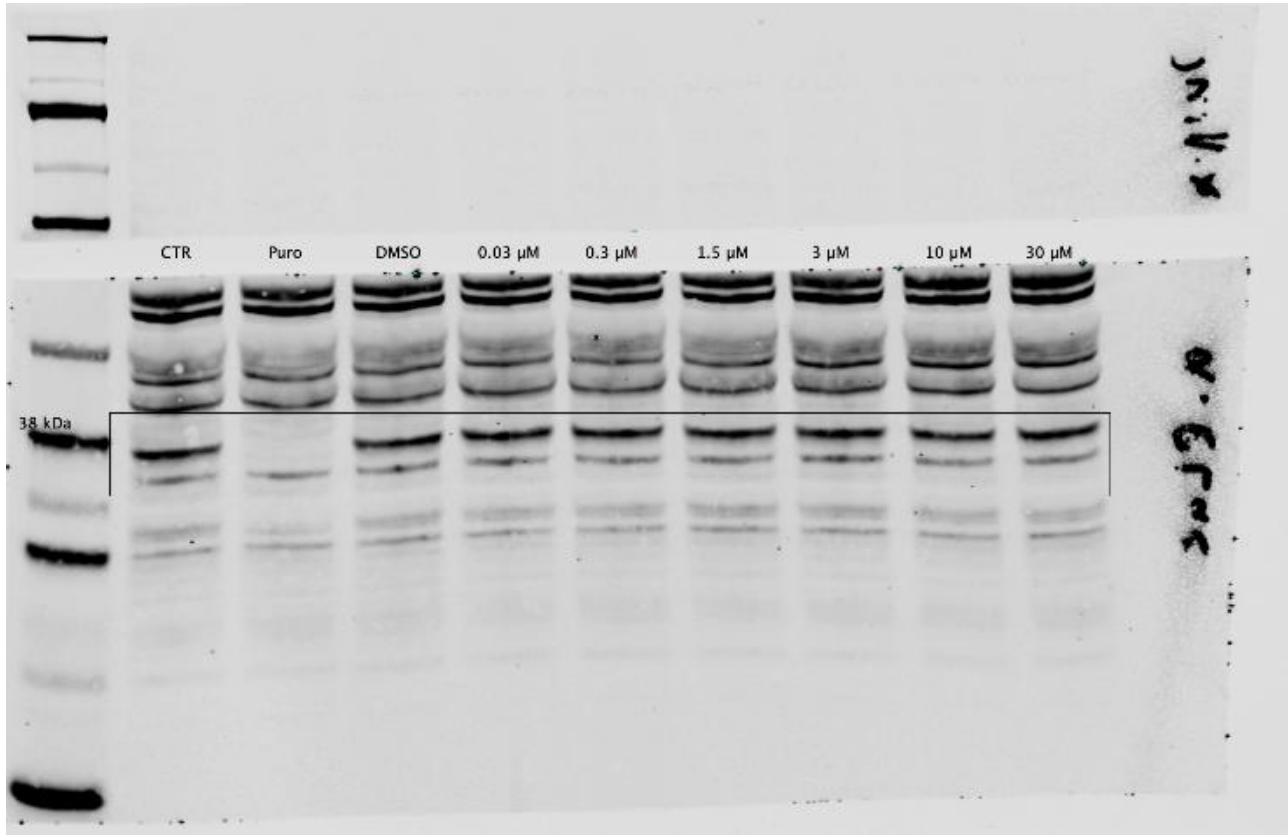

Sup. Figure 1A Vinculin

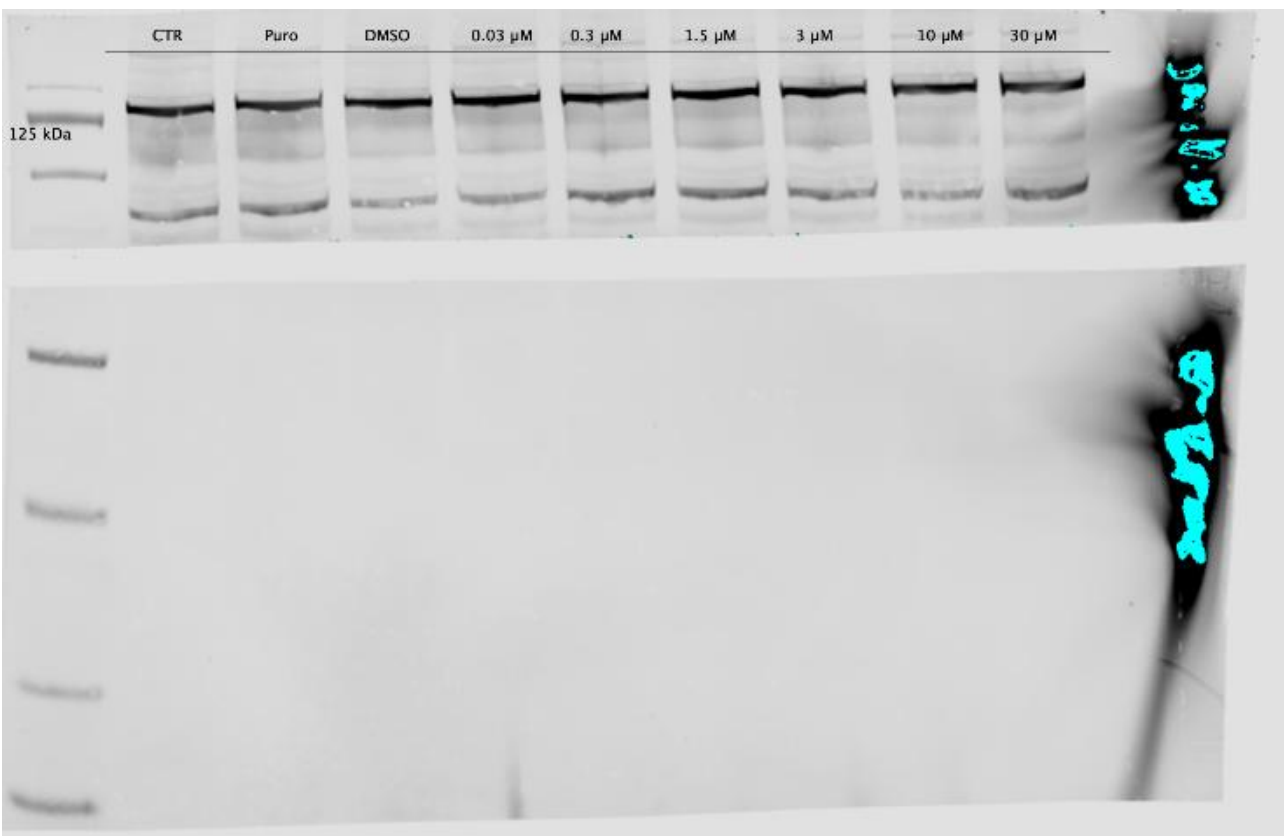

Sup. Figure 2A Puromycin

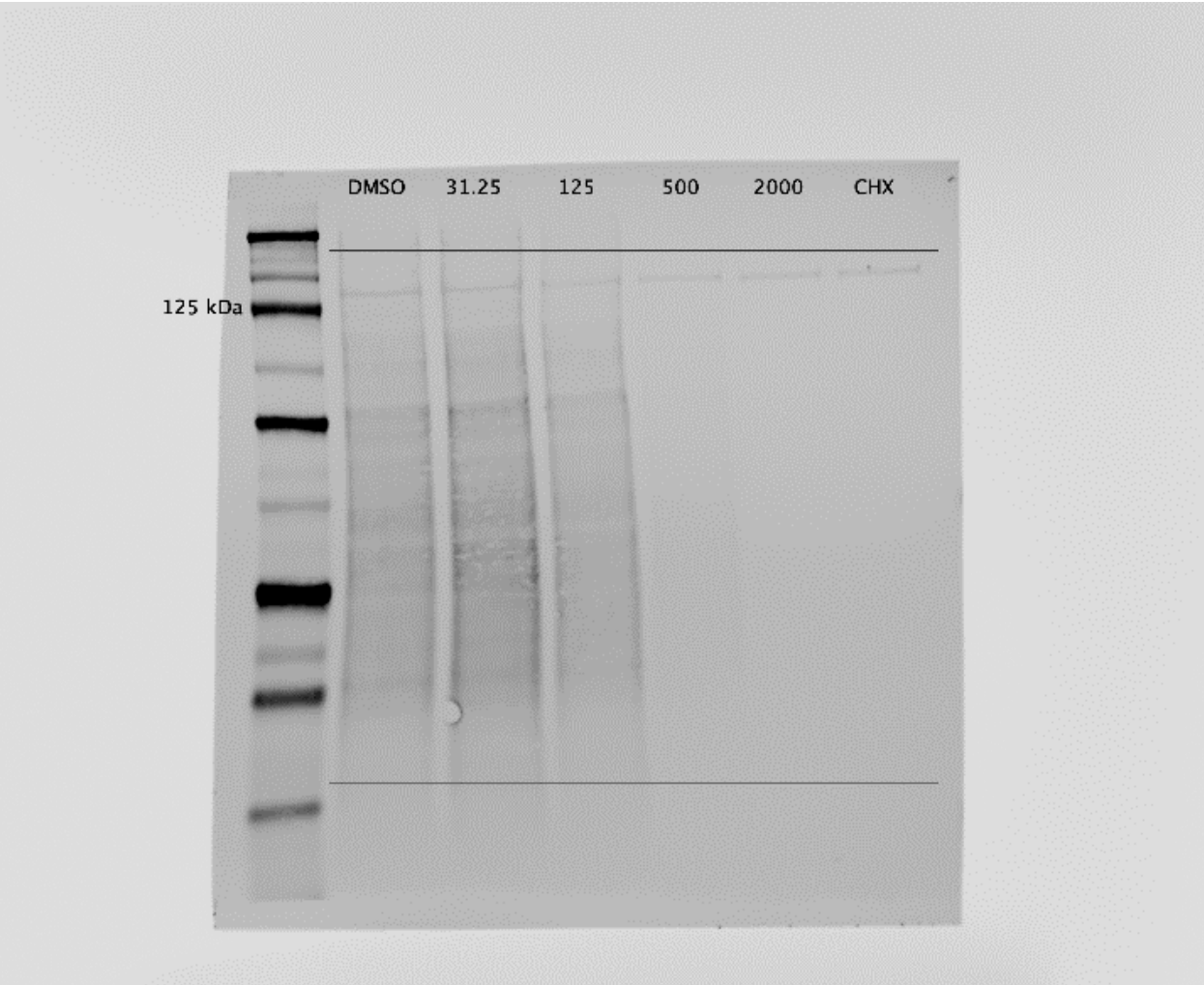

Sup. Figure 2A Vinculin

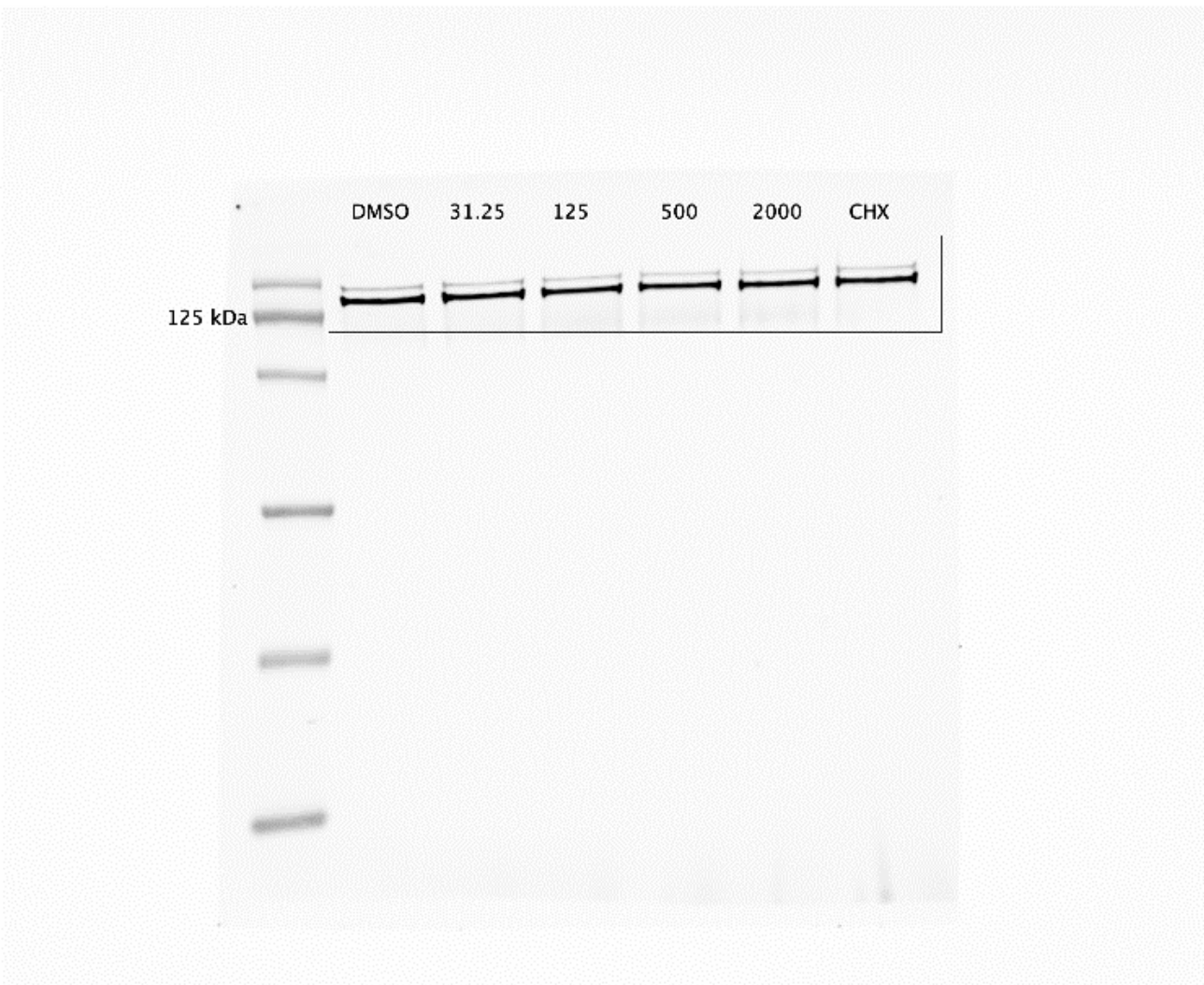

Supplement: Supplementary file 2 [file 42003_2026_10743_MOESM2_ESM.pdf]
