# Supplementary material for: A human cell-free translation screen identifies the NT-2 mycotoxin as a ribosomal inhibitor that binds the peptidyl transferase center
Source: Commun Biol. 2026 Sep 23;9:1237. doi: 10.1038/s42003-026-10743-6 (PMC13601500; doi:10.1038/s42003-026-10743-6)
Supplement: Supplementary file 3 [file 42003_2026_10743_MOESM3_ESM.pdf]

Description of Additional Supplementary Files

Supplementary Data 1

Numerical source data underlying the graphs in the main and Supplementary Figures, together with the screening hit list and validation outcomes. The file contains the values used to generate the plotted data, including replicate values where applicable, as well as compound identifiers, suppliers, library information, hit scores, IC50 values and validation classifications from the high-throughput screening workflow.
