# Supplementary material for: A human cell-free translation screen identifies the NT-2 mycotoxin as a ribosomal inhibitor that binds the peptidyl transferase center
Source: Commun Biol. 2026 Sep 23;9:1237. doi: 10.1038/s42003-026-10743-6 (PMC13601500; doi:10.1038/s42003-026-10743-6)
Supplement: Supplementary file 4 [file 42003_2026_10743_MOESM5_ESM.pdf]

nature portfolio

Corresponding author(s): Evangelos D. Karousis

Last updated by author(s): 2026.07.14

Reporting Summary

Nature Portfolio wishes to improve the reproducibility of the work that we publish. This form provides structure for consistency and transparency in reporting. For further information on Nature Portfolio policies, see our Editorial Policies and the Editorial Policy Checklist.

Statistics

For all statistical analyses, confirm that the following items are present in the figure legend, table legend, main text, or Methods section.

n/a	Confirmed	
☐	☒	The exact sample size (*n*) for each experimental group/condition, given as a discrete number and unit of measurement
☐	☒	A statement on whether measurements were taken from distinct samples or whether the same sample was measured repeatedly
☒	☐	The statistical test(s) used AND whether they are one- or two-sided<br>*Only common tests should be described solely by name; describe more complex techniques in the Methods section.*
☒	☐	A description of all covariates tested
☒	☐	A description of any assumptions or corrections, such as tests of normality and adjustment for multiple comparisons
☒	☐	A full description of the statistical parameters including central tendency (e.g. means) or other basic estimates (e.g. regression coefficient) AND variation (e.g. standard deviation) or associated estimates of uncertainty (e.g. confidence intervals)
☒	☐	For null hypothesis testing, the test statistic (e.g. *F*, *t*, *r*) with confidence intervals, effect sizes, degrees of freedom and *P* value noted<br>*Give P values as exact values whenever suitable.*
☒	☐	For Bayesian analysis, information on the choice of priors and Markov chain Monte Carlo settings
☒	☐	For hierarchical and complex designs, identification of the appropriate level for tests and full reporting of outcomes
☒	☐	Estimates of effect sizes (e.g. Cohen's *d*, Pearson's *r*), indicating how they were calculated

Our web collection on statistics for biologists contains articles on many of the points above.

Software and code

Policy information about availability of computer code

Data collection	*Provide a description of all commercial, open source and custom code used to collect the data in this study, specifying the version used OR state that no software was used.*
Data analysis	*Provide a description of all commercial, open source and custom code used to analyse the data in this study, specifying the version used OR state that no software was used.*

For manuscripts utilizing custom algorithms or software that are central to the research but not yet described in published literature, software must be made available to editors and reviewers. We strongly encourage code deposition in a community repository (e.g. GitHub). See the Nature Portfolio guidelines for submitting code & software for further information.

Data

Policy information about availability of data

All manuscripts must include a data availability statement. This statement should provide the following information, where applicable:

- Accession codes, unique identifiers, or web links for publicly available datasets
- A description of any restrictions on data availability
- For clinical datasets or third party data, please ensure that the statement adheres to our policy

The cryo-EM density maps and atomic coordinates have been deposited in the Electron Microscopy Data Bank (EMDB) and the Protein Data Bank (PDB) under accession codes EMD-57201/PDB 29IW, EMD-57202/PDB 29IX, EMD-57203/PDB 29IY and EMD-57204/PDB 29IZ. Numerical source data underlying the graphs in

the main and Supplementary Figures, together with the screening hit list and validation outcomes, are provided as Supplementary Data 1. Uncropped Western blot images are included in the Supplementary Information. All other data supporting the findings of this study are available from the corresponding author upon reasonable request.

Research involving human participants, their data, or biological material

Policy information about studies with human participants or human data. See also policy information about sex, gender (identity/presentation), and sexual orientation and race, ethnicity and racism.

Reporting on sex and gender	*Use the terms sex (biological attribute) and gender (shaped by social and cultural circumstances) carefully in order to avoid confusing both terms. Indicate if findings apply to only one sex or gender; describe whether sex and gender were considered in study design; whether sex and/or gender was determined based on self-reporting or assigned and methods used. Provide in the source data disaggregated sex and gender data, where this information has been collected, and if consent has been obtained for sharing of individual-level data; provide overall numbers in this Reporting Summary. Please state if this information has not been collected. Report sex- and gender-based analyses where performed, justify reasons for lack of sex- and gender-based analysis.*
Reporting on race, ethnicity, or other socially relevant groupings	*Please specify the socially constructed or socially relevant categorization variable(s) used in your manuscript and explain why they were used. Please note that such variables should not be used as proxies for other socially constructed/relevant variables (for example, race or ethnicity should not be used as a proxy for socioeconomic status). Provide clear definitions of the relevant terms used, how they were provided (by the participants/respondents, the researchers, or third parties), and the method(s) used to classify people into the different categories (e.g. self-report, census or administrative data, social media data, etc.) Please provide details about how you controlled for confounding variables in your analyses.*
Population characteristics	*Describe the covariate-relevant population characteristics of the human research participants (e.g. age, genotypic information, past and current diagnosis and treatment categories). If you filled out the behavioural & social sciences study design questions and have nothing to add here, write "See above."*
Recruitment	*Describe how participants were recruited. Outline any potential self-selection bias or other biases that may be present and how these are likely to impact results.*
Ethics oversight	*Identify the organization(s) that approved the study protocol.*

Note that full information on the approval of the study protocol must also be provided in the manuscript.

Field-specific reporting

Please select the one below that is the best fit for your research. If you are not sure, read the appropriate sections before making your selection.

☒ Life sciences ☐ Behavioural & social sciences ☐ Ecological, evolutionary & environmental sciences

For a reference copy of the document with all sections, see nature.com/documents/nr-reporting-summary-flat.pdf

Life sciences study design

All studies must disclose on these points even when the disclosure is negative.

Sample size	No statistical methods were used to predetermine sample size. For biochemical and cell-free translation assays, each experiment was performed in independent biological replicates (typically n = 3–4), which is standard in the field and sufficient to demonstrate reproducibility of the observed effects.
Data exclusions	No data were excluded from the analyses. All collected measurements are reported.
Replication	All key findings, including the effects of NT-2 on translation inhibition in lysates and cells, were reproduced in at least three independent experiments with consistent results.
Randomization	Randomization was not applicable, as no animal or human subjects were involved and all biochemical assays were performed on homogeneous lysate preparations.
Blinding	Blinding was not applicable, as experimental conditions (compound identity and control treatments) were predefined by the assay design and data acquisition was automated and quantitative.

Reporting for specific materials, systems and methods

We require information from authors about some types of materials, experimental systems and methods used in many studies. Here, indicate whether each material, system or method listed is relevant to your study. If you are not sure if a list item applies to your research, read the appropriate section before selecting a response.

Materials & experimental systems

n/a	Involved in the study	
☐	☒	Antibodies
☐	☒	Eukaryotic cell lines
☒	☐	Palaeontology and archaeology
☐	☒	Animals and other organisms
☒	☐	Clinical data
☒	☐	Dual use research of concern
☒	☐	Plants

Methods

n/a	Involved in the study	
☒	☐	ChIP-seq
☒	☐	Flow cytometry
☒	☐	MRI-based neuroimaging

Antibodies

Antibodies used

Mouse anti-FLAG M2 (Sigma, Cat. No. F1804, 1:1000),
Mouse anti-Vinculin (Santa Cruz Biotechnologies, Cat. No. sc-73614, 1:2000),
Mouse anti-Puromycin (Millipore, Cat. No. MABE343, 1:15’000, IgG2a),
Mouse anti-GAPDH (Santa Cruz Biotechnology, Cat. No. sc-47724, 1:10’000, IgG1),
Mouse anti-eIF2α (Cell Signaling Technology, Cat. No. 2103, 1:1000),
Rabbit anti-phospho-eIF2α (Ser51) (Cell Signaling Technology, Cat. No. 9721, 1:1000).
Donkey anti-mouse 680LT (LI-COR, Cat. No. 926–68022, 1:10’000),
Donkey anti-rabbit 800CW (LI-COR, Cat. No. 926–32212, 1:10’000),
Goat anti-mouse IgG2a 800CW (LI-COR, Cat. No. 926-32351, 1:10’000),
Goat anti-mouse IgG1 680LT (LI-COR, Cat. No. 926-68050, 1:10’000),
Goat anti-mouse 680LT (LI-COR, Cat. No. 926-68020, 1:10’000).

Validation

All antibodies were validated by the manufacturers for use in Western blotting of human lysates. Expected molecular weights and signal specificity were confirmed in our experiments by comparison with control samples and by signal loss upon protein translation inhibition.

Eukaryotic cell lines

Policy information about cell lines and Sex and Gender in Research

Cell line source(s)

HeLa S3 cells (Sigma-Aldrich, ECACC, Cat. No. 87110901), HEK293 Flp-In T-REx cells (Invitrogen Cat. No. R78007), BHK-21 (Clone 13) from hamster (MERCK 85011433-1VL)

Authentication

HEK293T cells, BHK-21 and HeLa S3 cells were authenticated through STR profiling (Microsynth, Switzerland)

Mycoplasma contamination

All cell lines tested negative for mycoplasma contamination by PCR-based assay prior to use.

Commonly misidentified lines
(See ICLAC register)

No commonly misidentified cell lines listed in the ICLAC database were used in this study.

Animals and other research organisms

Policy information about studies involving animals; ARRIVE guidelines recommended for reporting animal research, and Sex and Gender in Research

Laboratory animals

For laboratory animals, report species, strain and age OR state that the study did not involve laboratory animals.

Wild animals

Provide details on animals observed in or captured in the field; report species and age where possible. Describe how animals were caught and transported and what happened to captive animals after the study (if killed, explain why and describe method; if released, say where and when) OR state that the study did not involve wild animals.

Reporting on sex

Indicate if findings apply to only one sex; describe whether sex was considered in study design, methods used for assigning sex. Provide data disaggregated for sex where this information has been collected in the source data as appropriate; provide overall numbers in this Reporting Summary. Please state if this information has not been collected. Report sex-based analyses where performed, justify reasons for lack of sex-based analysis.

Field-collected samples

For laboratory work with field-collected samples, describe all relevant parameters such as housing, maintenance, temperature, photoperiod and end-of-experiment protocol OR state that the study did not involve samples collected from the field.

Ethics oversight

Identify the organization(s) that approved or provided guidance on the study protocol, OR state that no ethical approval or guidance was required and explain why not.

Note that full information on the approval of the study protocol must also be provided in the manuscript.

Plants

Seed stocks	*Report on the source of all seed stocks or other plant material used. If applicable, state the seed stock centre and catalogue number. If plant specimens were collected from the field, describe the collection location, date and sampling procedures.*
Novel plant genotypes	*Describe the methods by which all novel plant genotypes were produced. This includes those generated by transgenic approaches, gene editing, chemical/radiation-based mutagenesis and hybridization. For transgenic lines, describe the transformation method, the number of independent lines analyzed and the generation upon which experiments were performed. For gene-edited lines, describe the editor used, the endogenous sequence targeted for editing, the targeting guide RNA sequence (if applicable) and how the editor was applied.*
Authentication	*Describe any authentication procedures for each seed stock used or novel genotype generated. Describe any experiments used to assess the effect of a mutation and, where applicable, how potential secondary effects (e.g. second site T-DNA insertions, mosiacism, off-target gene editing) were examined.*
